# *Bacteroides uniformis* CECT 7771 Ameliorates Metabolic and Immunological Dysfunction in Mice with High-Fat-Diet Induced Obesity

**DOI:** 10.1371/journal.pone.0041079

**Published:** 2012-07-26

**Authors:** Paola Gauffin Cano, Arlette Santacruz, Ángela Moya, Yolanda Sanz

**Affiliations:** Microbial Ecology and Nutrition Research Group, Institute of Agrochemistry and Food Technology (IATA), National Research Council (CSIC), Valencia, Spain; Charité-University Medicine Berlin, Germany

## Abstract

**Background:**

Associations have been made between obesity and reduced intestinal numbers of members of the phylum Bacteroidetes, but there is no direct evidence of the role these bacteria play in obesity. Herein, the effects of *Bacteroides uniformis* CECT 7771 on obesity-related metabolic and immune alterations have been evaluated.

**Methods and Findings:**

Adult (6–8 week) male wild-type C57BL-6 mice were fed a standard diet or a high-fat-diet HFD to induce obesity, supplemented or not with *B. uniformis* CECT 7771 for seven weeks. Animal weight was monitored and histologic, biochemical, immunocompetent cell functions, and features of the faecal microbiota were analysed after intervention. The oral administration of *B. uniformis* CECT 7771 reduced body weight gain, liver steatosis and liver cholesterol and triglyceride concentrations and increased small adipocyte numbers in HFD-fed mice. The strain also reduced serum cholesterol, triglyceride, glucose, insulin and leptin levels, and improved oral tolerance to glucose in HFD fed mice. The bacterial strain also reduced dietary fat absorption, as indicated by the reduced number of fat micelles detected in enterocytes. Moreover, *B. uniformis* CECT 7771 improved immune defence mechanisms, impaired in obesity. HFD-induced obesity led to a decrease in TNF-α production by peritoneal macrophages stimulated with LPS, conversely, the administration of *B. uniformis* CECT 7771 increased TNF-α production and phagocytosis. Administering this strain also increased TNF-α production by dendritic cells (DCs) in response to LPS stimulation, which was significantly reduced by HFD. *B. uniformis* CECT 7771 also restored the capacity of DCs to induce a T-cell proliferation response, which was impaired in obese mice. HFD induced marked changes in gut microbiota composition, which were partially restored by the intervention.

**Conclusions:**

Altogether, the findings indicate that administration of *B. uniformis* CECT 7771 ameliorates HFD-induced metabolic and immune dysfunction associated with intestinal dysbiosis in obese mice.

## Introduction

Obesity is considered a major health issue due to its increasing prevalence and associated co-morbidities (e.g. type 2 diabetes, fatty liver and cardiovascular disease) affecting both the developed and the developing world [1]–[2]. This disorder is the result of a long-term positive energy imbalance and is associated with a chronic state of low grade inflammation and immune dysfunction [3]–[5].

The microbiota of the human gastrointestinal tract has been considered as an organ that contributes to human physiological diversity by encoding additional metabolic capacities and regulating gene expression of pathways involved in host nutrient metabolism [6]. Animal studies indicate that colonisation of adult germ-free mice with a distal gut microbial community harvested from conventionally raised mice influences energy-balance by increasing both nutrient digestion and absorption, and adiposity [7]. This effect is mediated by different mechanisms, including microbial fermentation of dietary polysaccharides that are otherwise indigestible by the host, subsequent intestinal absorption of monosaccharides and short-chain fatty acids, conversion of these metabolites to more complex lipids in the liver; and microbial regulation of host genes that promote lipid accumulation in adipocytes [8].

In humans, the gut microbiota composition is also thought to play a role in energy balance, as inferred from observational studies reporting associations between obesity or weight loss and shifts in gut microbiota composition. It has been reported that 80–90% of bacterial phylotypes are members of two phyla, namely Bacteroidetes (including the genera *Bacteroides* and *Prevotella)* and Firmicutes (including the genera *Clostridium, Enterococcus, Lactobacillus* and *Ruminococcus*), followed by the phyla Actinobacteria (including the genus *Bifidobacterium*) and Proteobacteria (including *Helicobacter* and *Escherichia*) [9]–[10]. Therefore, relative proportions of Firmicutes and Bacteroides are considered good indicators of major changes in intestinal microbiota constitution. So far, most studies have reported associations between obesity and reduced proportions of the phylum Bacteroidetes [11] which, in some cases, were accompanied by increased proportions of Actinobacteria [6] or Firmicutes [12], as also observed in mice [9], [13]. In addition, weight loss in obese human subjects subjected to dietary or surgical interventions has been associated with increases in Bacteroidetes [12] or in *Bacteroides* spp., *Bacteroides-Prevotella* spp. or *Bacteroides fragilis* group [14]–[17]. Nevertheless, few observational studies have found no associations between weight-loss or obesity and numbers of *Bacteroides* spp. or *Bacteroides-Prevotella* spp. [18]–[19], reported opposite associations between obesity, and the relative proportion of Bacteroidetes to Firmicutes [19] or the numbers of *Prevotellaceae*, a subgroup of Bacteroidetes [20]. These discrepancies could partly be explained by differences in the analytical techniques used since, for example, real-time PCR or FISH target only specific groups of Bacteroidetes and underestimate some members of this phylum present in faecal samples [21]. By contrast, DNA sequence analyses of the phylum Bacteroidetes can cover up to 46 species of bacteria belonging to seven different genera of the order *Bacteroidales* [6], encompassing a taxonomic unit that is too broad to assess its possible implication in obesity [21].

In this study, we hypothesised that modification of the gut microbiota structure by increasing numbers of specific *Bacteroides* spp. could contribute to restoring obesity-related metabolic and immune dysfunction. To obtain direct evidence of the role of *Bacteroides* spp. in obesity and thus confirm such a hypothesis, we have evaluated *in vitro* the immunologic properties of different intestinal *Bacteroides* spp. on macrophages. Furthermore, we have assessed the effect of the selected strain (*B. uniformis* CECT 7771) on metabolic and immune parameters of mice with high-fat-diet induced obesity.

## Methods

### Bacterial Strain and Culture Conditions

The following *Bacteroides* spp. and strains from the IATA-CSIC and Spanish Culture Collection (CECT) were studied: *Bacteroides dorei* SS1, *Bacteroides ovatus* SU2, *Bacteroides distasonis* CAY3, *Bacteroides uniformis* CECT 7771 (or CY1), *Bacteroides thetaiotaomicron* SAC4, *Bacteroides fragilis* SX3, *Bacteroides caccae* SV3 and *Bacteroides finegoldii* SX2. These strains were isolated from stools of healthy infants (mean age 5.7 years, range 1.0–10.8 years). Briefly, fresh stool samples were diluted (1∶10 [w/v]) in phosphate-buffered saline (PBS) solution (130 mM sodium chloride, 10 mM sodium phosphate, pH 7.2) and homogenised. Then, aliquots of serial dilutions in PBS and aliquots were plated on Schaedler Agar (Scharlau, Barcelona, Spain) supplemented with kanamycin (100 mg/L), vancomycin (7.5 mg/L) and vitamin K (0.5 mg/L) and incubated under anaerobic conditions at 37 °C for 48 hours. Individual colonies were isolated from the highest dilution plate from each subject and their cellular morphology and Gram-staining characteristics were examined. The isolated clones were identified at species level by sequencing of amplified 16S rDNA regions with the primers 27f and 1401r as previously described [22]. The PCR products obtained were purified using the GFXtm PCR DNA and Gel Band DNA Purification Kit (GE Healthcare, Buckinghamshire, UK) for DNA sequencing. DNA sequencing was carried out by an *ABI PRISM-3130XL Genetic Analyser* (Applied Biosystems, California, USA). The closest relatives of the partial 16S rRNA gene sequences were sought in GenBank using the Basic Local Alignment Search Tool (BLAST) algorithm, and sequences with more than 97% similarity were considered as belonging to the same species. All new data has been deposited in GenBank (Accession numbers: JX183979, JX262250, JX183978, JX183977, JX183984, JX183983, JX183981 and JX183982, respectively).

For experimental purposes, the bacterial strains were grown in Brain Heart Infusion Broth (BH) (Scharlab, SL- Barcelona, Spain) at 37 °C in microaerophilic conditions (AnaeroGen; Oxoid, Basingstoke, UK). Cells were harvested by centrifugation (6,000 g for 15 min), washed twice in phosphate buffered saline (PBS, 130± sodium chloride, 10 mM sodium phosphate, pH 7.4), and re-suspended in PBS plus 15% glycerol for *in vitro* trials and in 10% skimmed milk for animal trials. Aliquots of these suspensions were frozen in liquid nitrogen and stored at -80°C until used. The number of live cells after freezing and thawing was determined by colony-forming unit (CFU) counting on BH agar after 48 h incubation. For the strain tested, more than 90% cells were alive upon thawing and no significant differences were found during storage time (2 months). One fresh aliquot was thawed for every new experiment to avoid variability in the viability of cultures.

### Effect of *Bateroides* Strains on Induction of Cytokine Production by Macrophages

To evaluate the immunological properties of different *Bacteroides* strains, the RAW 264 macrophage cell line, obtained from the American Type Culture Collection (Rockville, MD, USA), was cultured overnight in 24-well flat-bottom polystyrene microtiter plates (Corning, Cultek, Madrid, Spain) at a concentration of 1×10^5^ cells per ml in Dulbeco’s Modified Eagles Medium (DMEM) (SigmaTM– St. Louis, MO/USA). Media were changed before stimulation and, then, cells were incubated in the presence of 100 µl of a cell suspension (1×10^7^ cfu/ml) of each *Bacteroides* strain for 24 h. Purified LPS from *Salmonella enterica* serotype Typhimurium (Sigma Chemical Co, Madrid, Spain) was used at a concentration of 1 µg/ml as a positive control. Non-stimulated Raw 264.7 cells were also evaluated as controls of basal cytokine production. The cell culture supernatants were collected and stored at −20°C until used for cytokine determination. TNF-α and IL-10 were quantified by ELISA Ready SET Go! Kit (BD Bioscience, San Diego, CA, USA). Every parameter was assayed in triplicate in two independent experiments.

**Table 1:** Oligonucleotide primers used in this study.

Targetbacterial groups	Primers(name)	Sequence (5′–3′)	Size(pb)	AnnealingTmp (°C)	References
Total bacteria	HDA 1,HDA 2	TGGCTCAGGACGAACGCTGGCGGC CCTACTGCTGCCTCCCGTAGGAGT	200	59	(26)
*Bifidobacterium* spp.	BiFid F,BiFid R	CTCCTGGAAACGGGTGG GGTGTTCTTCCCGATATCTACA	550	55	(26,27)
*Bacteroides* spp.	Bfra F,Bfra R	ATA GCC TTT CGA AAG RAA GATCCA GTA TCA ACT GCA ATT TTA	287	55	(26,27)
*Clostridium coccoides* group	Ccoc F,Ccoc R	AAA TGA CGG TAC CTG ACT AA CTT TGA GTT TCA TTC TTG CGA A	440	50	(26,27)
*Clostridium leptum* group	ClepF,Clep R3	GCA CAA GCA GTG GAG T CTT CCT CCG TTT TGT CAA	239	50	(26,27)
*Enterobacteriaceae*	Entero 1,Entero 2	CATTGACGTTACCCGCAGAAGAAGC CTCTACGAGACTCAAGCTTGC	195	63	(28)
*Lactobacillus* group	Lac 1,Lac 2	AGCAGTAGGGAATCTTCCAATTYCACCGCTACACATG	340	61	(29, 30)

### Animals, Diets and Experimental Design

Animal experiments were carried out in strict accordance with the recommendations in the Guide for the Care and Use of Laboratory Animals of University of Valencia (Central Service of Support to Research [SCSIE], University of Valencia, Spain) and the protocol was approved by its Ethic Committee (approval ID A1245740259386). Adult (age 6–8 week) male wild-type C57BL-6 mice were purchased from Harlan Laboratories. During the adaptation period (7 days), six animals were housed in each stainless-steel cage in a temperature-controlled (23 °C) room with a 12-h light/dark cycle and 40–50% relative humidity. Then, mice were randomly divided into four groups (n = 6−8 mice per group) as follows: (1) a control group, receiving a standard diet (SD); (2) an obese group, receiving a high-fat diet (HFD); (3) a group receiving a SD and a daily dose of 5.0×10^8^ CFU *B. uniformis* CECT 7771 by gavage; and (4) an obese group receiving the HFD and a daily dose of 5.0×10^8^ CFU *B. uniformis* CECT 7771 by oral gavage. To induce obesity, mice were switched from the SD (CA.170481-AIN-76A Purified Diet-Rats/Mice, Harlan Laboratories, Madison, WI 53744-4220) administered during the adaptation period to all mice, to a HFD (TD.06414 - Adjusted Calories Diet - 60/Fat, Harlan Laboratories, Madison, WI 53744-4220) for 7 weeks. The HFD provided 18.4% kcal as protein, 21.3% kcal as carbohydrate and 60.3% kcal as fat (5.1 kcal/g), whereas the SD provided 18.8% kcal as protein, 68.8% kcal as carbohydrate and 12.4% kcal as fat (3.8 kcal/g). Therefore, there was an increase in fat at expenses of a reduction in carbohydrates in the HFD. Mice had free access to feed and sterile water.

Body weight was measured once a week and, at the end of study, animals were fasted for 16 h, anaesthetised, bled by aortic puncture and sacrificed by cervical dislocation. To analyse the metabolic parameters, blood samples were collected in tubes containing EDTA and centrifuged to obtain plasma that was stored at -20°C. Stools were collected at the end of the experimental period (7 weeks) for microbiological analyses. The liver, white adipose (perirenal and epididymal) and small intestinal tissues were excised and rinsed with saline solution, and sections were fixed in 10% neutral formalin buffered solution for histological analysis. The white adipose deposits (perirenal and epididymal) were previously weighed. Liver sections were also used for lipid extraction and quantification as described below.

### Histology of Liver, White Adipose Tissues and Small Intestine

Paraffin-embedded tissues were sectioned to a thickness of 4–5 µm and fixed to glass slides. Slides were deparaffinised and stained with haematoxylin-eosin. The severity of steatosis was determined in 100 hepatocytes of two liver tissue sections per mouse and scored as follows: grade 0 when fat was not detected in hepatocytes; grade 1 when fat occupied less than 30% of hepatocytes; grade 2 when fat occupied between 30 and 60% of hepatocytes; grade 3, when fat occupied more than 60% of hepatocytes.

Adipocyte cell sizes were measured in 100 cells of two sections of epididymal adipose tissue per mouse [23]. Adipocyte cell sizes were expressed as area ranges using the following ranges: <2000, 2000–4000,4000–6000 and 6000–7000 µm^2^.

The ratio of fat micelles to enterocyte was determined in 100 cells from two sections of small intestinal tissue of each mouse by counting ten 100X light microscope fields. All parameters were measured in a NIKON Eclipse 90i Microscopic, using the NIS Elements BR 2.3 basic research software (Kingston, Surrey, KT2 5PR, England). All histological analyses were conducted by an experienced histologist in a blind fashion.

### Analysis of Serum and Liver Biochemical Parameters and Glucose Tolerance

Biochemical parameters were quantified in plasma using enzymatic assay kits for glucose (Glucose Liquid Kit; Química Analítica Aplicada SA, Spain), cholesterol (Cholesterol Liquid kit, Química Analítica Aplicada SA, Spain) and triglycerides (Triglyceride Liquid kit, Química Analítica Aplicada SA, Spain). Serum leptin concentration was determined by enzyme-linked immunosorbent assay (ELISA) (BD Bioscience, San Diego, CA, USA). Serum insulin was determined by Ultrasensitive Mouse Insulin ELISA (Mercondia AB, Sweden, 2010).

Triglycerides and cholesterol were also quantified in liver. Lipids were extracted by homogenising the tissue with 2∶1 chloroform-methanol (v/v) making a 20-fold dilution, and filtering the homogenate through a nylon filter (Cell Strainer 40 micrometros Nylon.BD Falcon; BD BIOSCIENCIES). Non-lipidic substances were eliminated by adding 5-volumes of CaCl_2_ solution (5 mg/L) in water to the filtrate fraction. After centrifugation (4,000 g, for 5 minutes, at room temperature) the upper phase, containing all of the non-lipidic substances, was discarded. This process was repeated three times. Extracted lipids were dried under vacuum (Concentrator Plus 5301, Eppendorf Inc., NY, USA) and then triglyceride and total cholesterol concentrations were determined as described above.

Oral glucose tolerance tests were performed *in vivo* after 6 weeks of treatment. Food was removed 2 h after the onset of the daylight cycle and, after a 4-h fasting period, glucose was administered orally at a dose of 2 g/kg and blood samples were taken with heparinised capillary tubes from the tail vein before and 15, 30, 60, 90 and 120 minutes after glucose administration. Plasma glucose levels were analysed with glucose test strips (Ascensia Esyfill, Bayer, Tarrytown, NY; USA) and a glucometer (Ascensia VIGOR, Bayer Tarrytown, NY; USA), with a detection level ranging from 30 to 550 mg glucose/dl.

**Table 2:** Effect of different *Bacteroides* strains on cytokine production by RAW264.7 macrophages.

*Bacteroides* strains	Cytokine production	
	TNF-α (pg/ml)	IL-10 (pg/ml)
DEMEN	491.2(112.1)^a, b’^	97.2(10.8)^a,a’^
LPS	1425.4(77.6)^b, a’^	162.3(37.6)^a,a’^
*B. dorei* SS1	3765.5(150.0)^b,b’,a’’^	215.8(12.5)^b,a’,b’’^
*B. ovatus* SU2	4515.7(211.3)^b,b’,b’’^	271.5(8.1)^b,b’,b’’^
*B. distasonis* CAY3	4462.4(173.9)^ b,b’,b’’^	215.8(9.7)^b,a’,b’’^
*B. uniformis* CECT 7771	2998.4(50.4)^ b,b’,a’’^	341.3(13.5)^b,b’,a’’^
*B. thetaiotaomicron* SAC4	2931.2(464.5)^ b,b’,a’’^	109.2(3.0)^a,a’,b’’^
*B. fragilis* SX3	6657.3(278.3)^ b,b’,b’’^	81.2(14.6)^a,a’,b’’^
*B. caccae* SV3	11622.0(818.3)^ b,b’,b’’^	171.7(12.9)^b,a’,b’’^
*B. finegoldii* SX2	6535.8(62.2)^ b,b’,b’’^	83.5(17.4)^a,a’,b’’^

Purified lipopolysaccharide (1 mg/ml) (LPS) from *S. enterica* serotype Typhimurium was used as a positive control. Non-stimulated cells were also evaluated as controls of basal cytokine levels (DEMEN). Results are expressed as mean (SD) of duplicate measures determined in three independent experiments. Significant differences were established at *P<*0.05 by applying ANOVA and *post hoc* Tukey’ test. Means in the same columns with different letters were significantly different in relation to non-stimulated cells (a-b) or to LPS (a’-b’) or to *B. uniformis* CECT 7771 (a’’-b’’).

### Isolation and Assessment of Cytokine Production by Peritoneal Macrophages

Peritoneal cells were collected by washing the peritoneal cavity of different groups of mice, with 5 ml of sterile cold DMEM (Sigma), containing 10% inactivated (56°C for 30 min) foetal bovine serum (FBS) (Gibco, Barcelona, Spain), 100 µg/ml streptomycin and 100 U/ml penicillin (SigmaTM– St. Louis, MO/USA). Isolated macrophages were plated in flasks (Corning, Cultek, Madrid, Spain) at a concentration of 1×10^6^ cells per ml in DMEM and incubated for 2 h at 37°C in an atmosphere containing 5% CO_2_. Non-adhered cells were washed out with warm PBS. To evaluate differences in the response to a common stimulus, macrophages from different mouse groups were incubated in the presence of purified LPS from *Salmonella enterica* serotype Typhimurium (Sigma Chemical Co, Madrid, Spain) at a concentration of 1 µg/ml. Non-stimulated peritoneal macrophages were also evaluated as controls of basal cytokine production. To evaluate microbiota-related immune properties of faecal samples, macrophages from control mice were incubated in the presence of faecal samples (30 µl of 10-fold dilution) from the different mouse groups for 24 h. Stool samples used as stimuli were collected from six mice of each experimental group at the end of the study, diluted 10-fold in PBS and homogenised for 3 min. Macrophage culture supernatants were collected and stored at −20°C until used for cytokine determination. TNF-α and IL-10 were quantified by ELISA Ready SET Go! Kit (BD Bioscience, San Diego, CA, USA). Each parameter was assayed in triplicate in two independent experiments.

### Bactericidal Activity of Peritoneal Macrophages

The bactericidal activity of peritoneal macrophages was analysed according to Vieira *et al.* [24] Cells were washed with serum-free DMEM and nitroblue tetrazolium (NBT – SigmaTM– St. Louis, MO/USA) at 0.5 mg/ml together with a bacterial extract (Stimulant, No. 840-15-SigmaTM– St. Louis, MO/USA) in an equivalent concentration of McFarland Scale 2 in Lab-tek chamber slide w/cover (Nalge Nunc International, USA). After 1 h of incubation at 37 °C in 5% CO_2_ atmosphere, the cells were washed with PBS, then fixed with 4% paraformaldehyde and observed in a NIKON Eclipse 90i Microscope, using the NIS Elements BR 2.3 basic research software (Kingston, Surrey, KT2 5PR, England). One hundred cells were counted per mouse and the percentage of NBT positive cells was determined. This measurement was taken in triplicate, in two independent experiments.

### Isolation and Cytokine Production by Bone Marrow-derived Dendrite Cells (DC)

DCs were generated from bone marrow as described previously [25]. Cells were seeded at a concentration of 1×10^6^ (90–94% DCs) in 1 ml of culture medium without rm GM-CSF in 24-well plates (Corning, Cultek, Madrid, Spain) and incubated in the presence of faeces (30 µl) from the respective mouse group at 37°C under 5% CO_2_ for 24 h. Purified LPS from *S. enterica* serotype Typhimurium (Sigma Chemical Co, Madrid, Spain) was used at a concentration of 1 µg/ml as a positive control. Non-stimulated DCs were also evaluated as controls of basal cytokine production. Stool samples were obtained and prepared as described above. The cell culture supernatants were collected and stored at −20°C until used for cytokine determination (TNF-α and IL-10) as described above. Every parameter was assayed in triplicate, in two independent experiments.

### Interactions between DCs and CD4+ T Lymphocytes

CD4+ T lymphocytes were isolated from mouse spleens, which were excised, suspended in complete medium and passed through a stainless steel wire mesh. The obtained crude cell suspension was washed once. CD4+ T cells were immune-magnetically isolated by positive selection with “CD4+ (L3T4) microbeads” (Miltenyi Biotec GmbH, Bergisch Gladbach, Germany), following the manufacturer’s instructions. CD4+ T cells (purity exceeded 95%) were used for mixed lymphocyte reaction.

Isolated DCs were incubated for 24 h in the presence of 1 µg/ml LPS from *S.* Typhimurium (Sigma Chemical Co, Madrid, Spain). Aliquots of mature DCs from different mouse groups were plated in triplicate with allogeneic CD4+ T cells (TL) at 1∶1, 1∶2, and 1∶4 TL/DC cell ratios, in 0.2 ml culture medium in 96-well flat-bottomed plates (Corning, Cultek, Madrid, Spain) at 37 °C for 72 h. Lymphocyte proliferation was measured with the cell proliferation ELISA BrdU-colorimetric assay (Roche, Diagnostic, Germany). Individual cultures of DCs and TL stimulated with or without ConA, used as mitogen, were used as controls.

### Samples and Microbial Analysis by Quantitative PCR (qPCR)

Stool samples were weighed, diluted 1∶5 (w/v) in PBS (pH 7.2), homogenised by shaking in a vortex and stored at -20 °C till analysed. One aliquot of this dilution was used for DNA extraction using the QIAamp DNA stool Mini kit (Qiagen, Hilden, Germany). Specific primers (Table 1) [26]–[30] targeting different bacterial genera and species were used to characterise the composition of the microbiota by qPCR using LightCycler® 480 SYBR Green I Master (Roche, USA) with a an ABI PRISM 7000-PCR sequence detection system (Applied Biosystems, UK), as described previously [14].

### Statistical Analyses

Statistical analyses were carried out using SPSS 11.0 software (SPSS Inc., Chicago, IL, USA). Data of biochemical parameters were normally distributed and significant differences were determined by applying One-Way Anova with *pos hoc* Tukey’s test. Remaining data were non-normally distributed and the differences were determined by applying the Mann-Whitney *U* tests. In every case, *P-*values <0.05 were considered statistically significant.

## Results

### Bacteroides Strain Selection Based on in vitro Ability to Induce Cytokine Production

Results in Table 2 show the ability of different *Bacteroides* strains to induce cytokine production by Raw264.7 macrophages. All strains induced the production of significantly higher amounts of the pro-inflammatory cytokine TNF-α than the non-stimulated cells and LPS-stimulated macrophages, although the magnitude of this effect was strain-dependent. The strains *B. dorei* SS1, *B. uniformis* CECT 7771 and *B. thetaiotaomicron* SAC4 induced the lowest TNF-α production. Different bacterial strains also induced anti-inflammatory cytokine IL-10 production above basal levels to different extents, except for *B. thetaiotaomicron* SAC4, *B. fragilis* SX3 and *B. finegoldii* SX2 whose effects were not significant. *B. uniformis* CECT 7771 induced the highest IL-10 levels compared to the other *Bacteroides* strains under study. Therefore, this strain was selected for its greater anti-inflammatory properties.

### Body Weight Gain, Adipose Tissue Weight and Biochemical Parameters in Obese Mice

**Figure 1:**
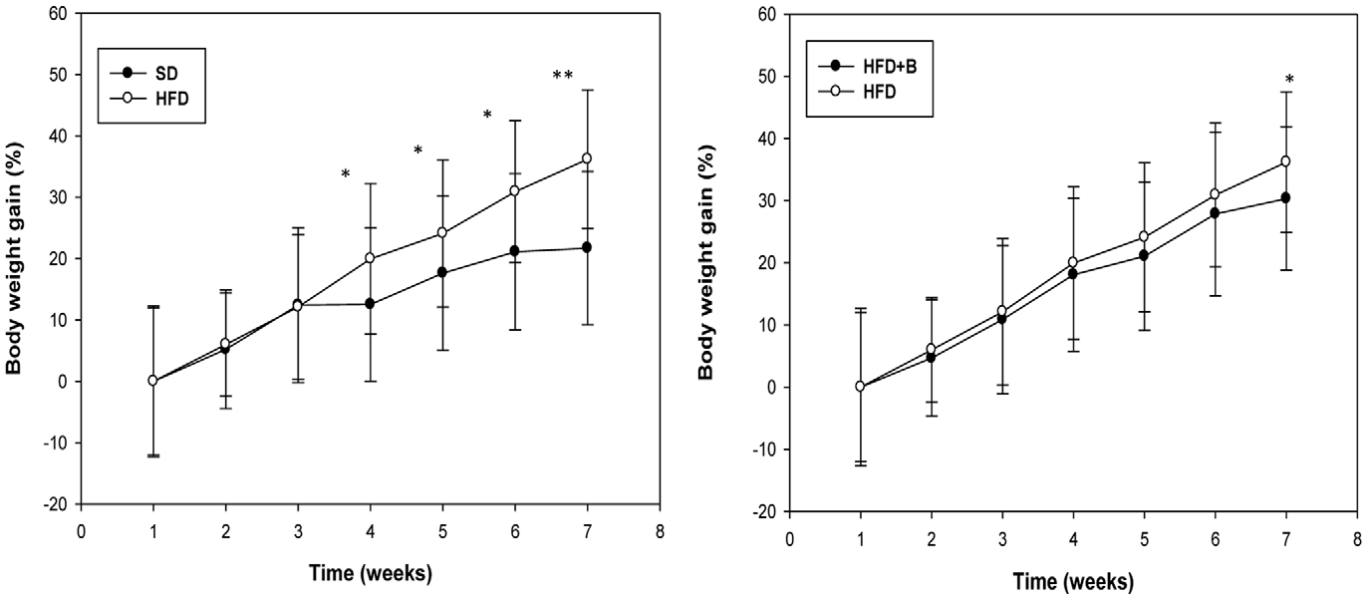
Time course of relative body weight gain in control mice and mice with high fat diet-induced obesity, administered or not *B. uniformis* CECT 7771. SD: standard diet group (control) (n = 6); HFD: high fat diet group (n = 6); HFD+B: high fat diet group receiving a daily dose of 5.0×10^8^ CFU *B. uniformis* CECT 7771 by gavage for 7 weeks (n = 6).

Body weight gain and total adipose tissue weight of mice after a 7-week intervention are shown in Table 3. The time course body weight gain over the intervention period is also shown in Figure 1. HFD-fed mice experienced significant greater weight gain compared to SD-fed mice from the first 4 weeks of treatment till the 7^th^ week. The weights of total adipose tissues were also statistically significantly greater in obese mice than in lean mice. The administration of *B. uniformis* CECT 7771 significantly reduced body weight gain in HFD-fed mice by the end of the intervention, but did not significantly modify total adipose tissue weight (Table 3, Figure 1). No mice died and all of them remained healthy throughout the study.

**Table 3:** Biometric parameters and serum and liver biochemistry in mice fed either a high-fat diet (HFD) or standard diet (SD), supplemented or not with *B. uniformis* CECT 7771.

Outcome measure	Experimental Groups										
	SD		HFD		SD+B		HFD+B		*P*- value HFD vsSD	*P*- value (SD+B vs SD)	*P*- value (HFD+B vs HFD)
	Mean	sd	Mean	sd	Mean	sd	Mean	sd			
**Biometric parameters**											
Body weight gain (%)	24.21	3.34	36.19	1.55	23.61	3.17	30.33	0.92	0.007*	0.890	0.005*
Adipose tissue (g)/100 gbody weight	0.06	0.04	0.15	0.03	0.03	0.02	0.14	0.04	0.016*	0.150	0.423
**Serum parameters**											
Cholesterol (mg/dl)	120.00	13.67	176.02	14.91	128.22	11.91	143.97	17.29	<0.001*	0.222	0.003*
Triglyceride (mg/dl)	130.31	11.56	156.99	27.47	129.77	13.94	118.21	10.04	0.041*	0.937	0.004*
Glucose (mg/dl)	219.81	26.41	485.92	140.63	372.41	13.50	233.52	30.62	0.001*	0.237	0.002*
Insulin (µg/l)	0.57	0.47	1.59	0.09	0.69	0.05	0.92	0.14	0.018*	0.892	0.018*
Leptin (ng/ml)	8.07	1.12	18.28	4.28	6.80	1.23	12.98	3.24	<0.001*	0.048*	0.014*
**Liver lipids**											
Cholesterol (mg/g )	29.94	4.08	35.51	4.35	29.22	6.32	27.48	6.39	0.029*	0.801	0.024*
Triglyceride (mg/g )	22.93	13.03	45.99	11.53	31.36	4.76	34.17	9.51	<0.001*	0.142	0.039*

SD: standard diet group (control) (n = 6); SD+B: standard diet group receiving a daily dose of 5.0×10^8^ CFU *B uniformis* CECT 7771 by gavage for 7 weeks (n = 6); HFD: high fat diet group (n = 6); HFD+B: high fat diet group receiving a daily dose of 5.0×10^8^ CFU *B. uniformis* CECT 7771 by gavage during 7 weeks (n = 6). Body composition and biochemical parameters were determined after 7 weeks of intervention. Adipose tissue included epididymal and perirenal white adipose tissues. Values are expressed as means and standard deviation (sd).

* Significant differences were established at *P<*0.050.

**Figure 2:**
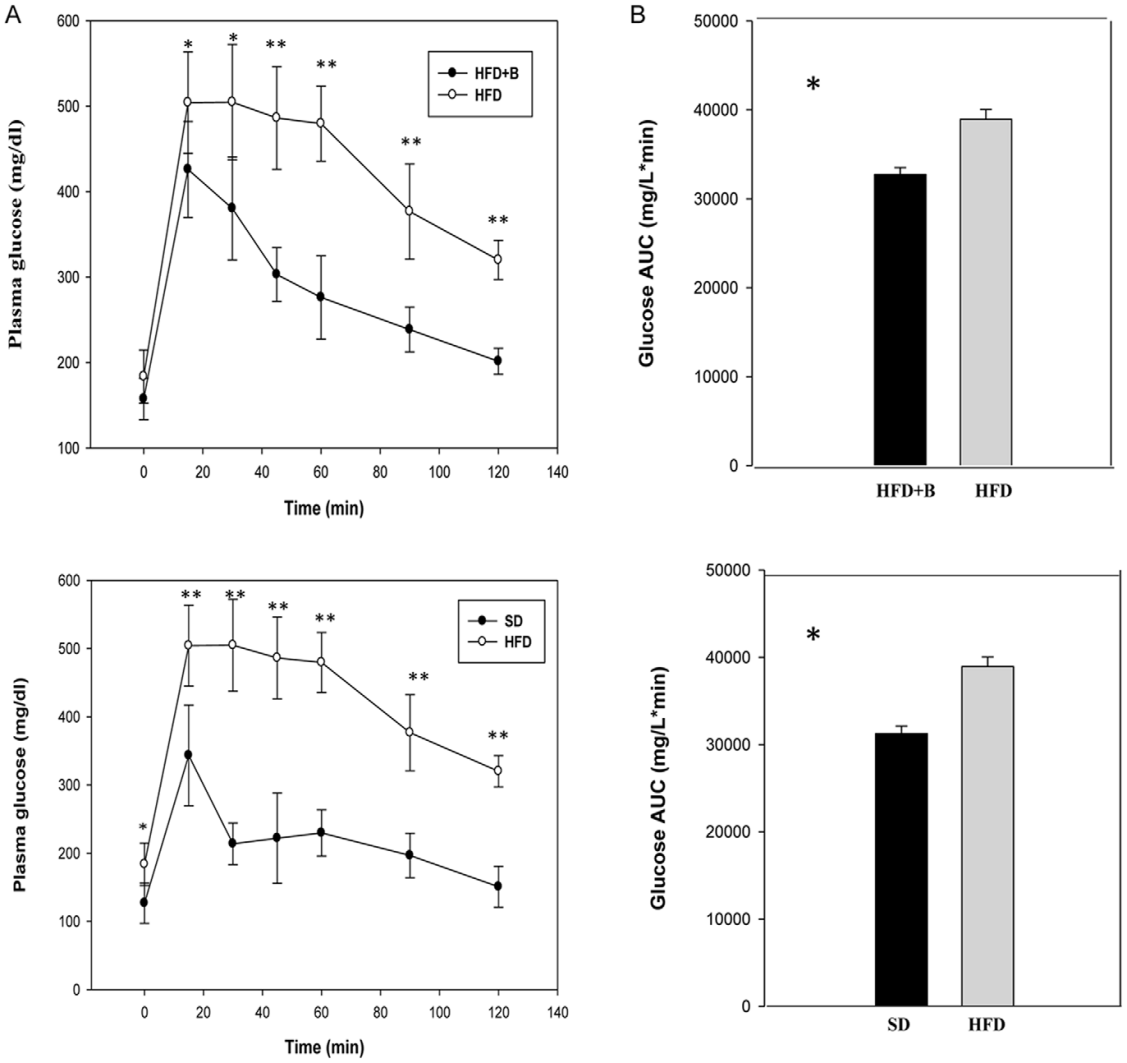
Glucose tolerance in control mice and mice with high fat diet-induced obesity, administered or not *B. uniformis* CECT 7771. SD: standard diet group (control) (n = 6); HFD: high fat diet group (n = 6); HFD+B: high fat diet group receiving a daily dose of 5.0×10^8^ CFU *B. uniformis* CECT 7771 by gavage for 7 weeks (n = 6). A: Plasma glucose profile following 2 g/kg glucose oral challenge after 4 h fasting; B: Mean area under the curve measured between 0 and 120 min after glucose administration.

Serum concentrations of biochemical and hormonal parameters of metabolic relevance are shown in Table 3. The HFD induced a significant increase in all serum parameters analysed compared to SD-fed mice. The administration of *B. uniformis* CECT 7771 significantly reduced serum glucose, insulin, triglycerides and cholesterol concentrations in HFD-fed mice but not in SD-fed mice. In HFD-fed mice *B. uniformis* CECT 7771 reduced cholesterol levels by 18% and triglyceride levels by 25%. *B. uniformis* CECT 7771 administration also significantly lowered fasting glucose levels (Table 3) and improved glucose tolerance, reducing the maximum peak and the area under the curve during the oral glucose tolerance test (Figure 2). The increased serum leptin concentrations induced by the HFD were significantly reduced by the administration of *B. uniformis* CECT 7771 (Table 3).

The specific concentrations of cholesterol and triglycerides present in total lipids extracted from the liver were also analysed (Table 3). The HFD induced a significant increase in both parameters compared to levels in SD-fed mice, while the administration of *B. uniformis* CECT 7771 significantly reduced triglycerides and cholesterol concentrations in the liver of HFD-fed mice (Table 3).

### Hepatic Steatosis, Adipocyte size, and Fat Absorption by Enterocytes in Obese Mice

**Figure 3:**
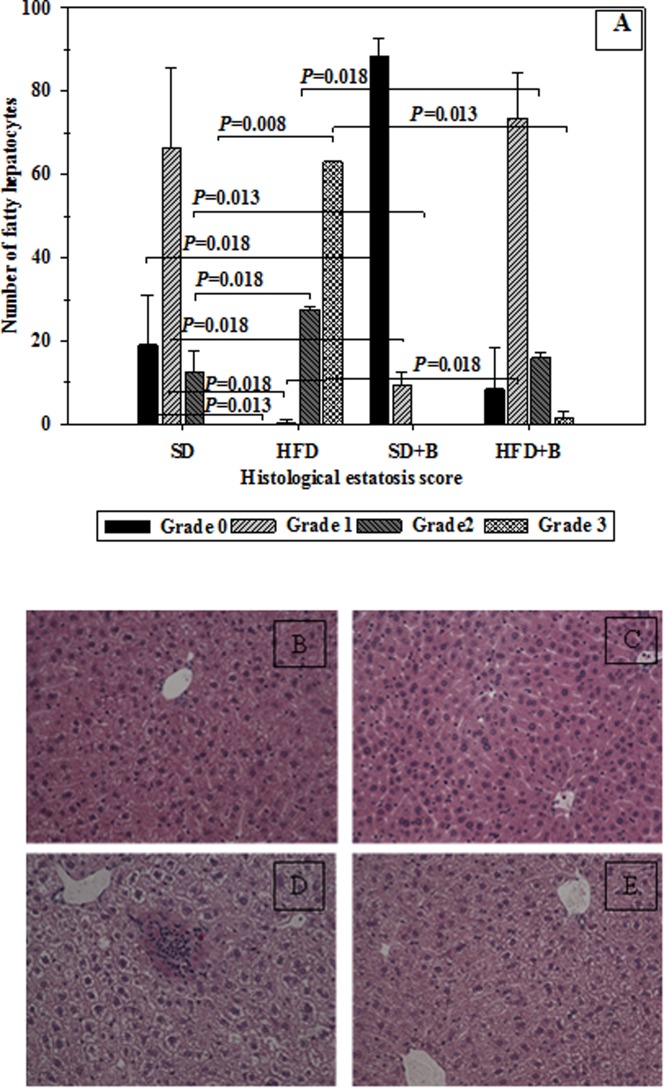
Determination of hepatic steatosis (hepatic histology) in control mice and mice with high fat diet-induced obesity, administered or not *B. uniformis* CECT 7771. SD: standard diet group (control) (n = 6); SD+B: standard diet group receiving a daily dose of 5.0×10^8^ CFU *B. uniformis* CECT 7771 by gavage for 7 weeks (n = 6); HFD: high fat diet group (n = 6); HFD+B: high fat diet group receiving a daily dose of 5.0×10^8^ CFU *B. uniformis* CECT 7771 by gavage for 7 weeks (n = 6).The fat vacuoles were measured in 100 hepatocytes of two liver tissue sections per mouse and scored for the severity of steatosis according to the following criteria: For grade-0 steatosis, no fatty hepatocytes; grade-1 steatosis, fat occupying less than 30% of the hepatocyte; grade-2 steatosis, fat occupying less than 30 to 60% of the hepatocyte; grade-3 steatosis, fat occupying more than 60% of the hepatocyte. Photomicrographs 20X of representative HE-stained slides are shown. (B) SD group, (C) SD+P group, (D) HFD group and (F) HFD+P group. Data are expressed as means ± SD and statistically significant differences are established at *P*<0.05.

Figure 3 shows the effects on hepatic steatosis of the administration of *B. uniformis* CECT 7771 to SD- or HFD-fed mice. The bacterial strain significantly reduced steatosis in HFD-fed animals, and also reduced lipid accumulation in the liver of SD-fed mice. The administration of *B. uniformis* CECT 7771 to SD mice significantly increased the number of hepatocytes with no steatosis (0-grade) and reduced those with steatosis grades 1 and 2. In HFD-fed animals, *B. uniformis* CECT 7771 administration contributed to increasing the number of hepatocytes with 0- and 1-grade steatosis and to reducing those with 2-and 3-grade steatosis.

**Figure 4:**
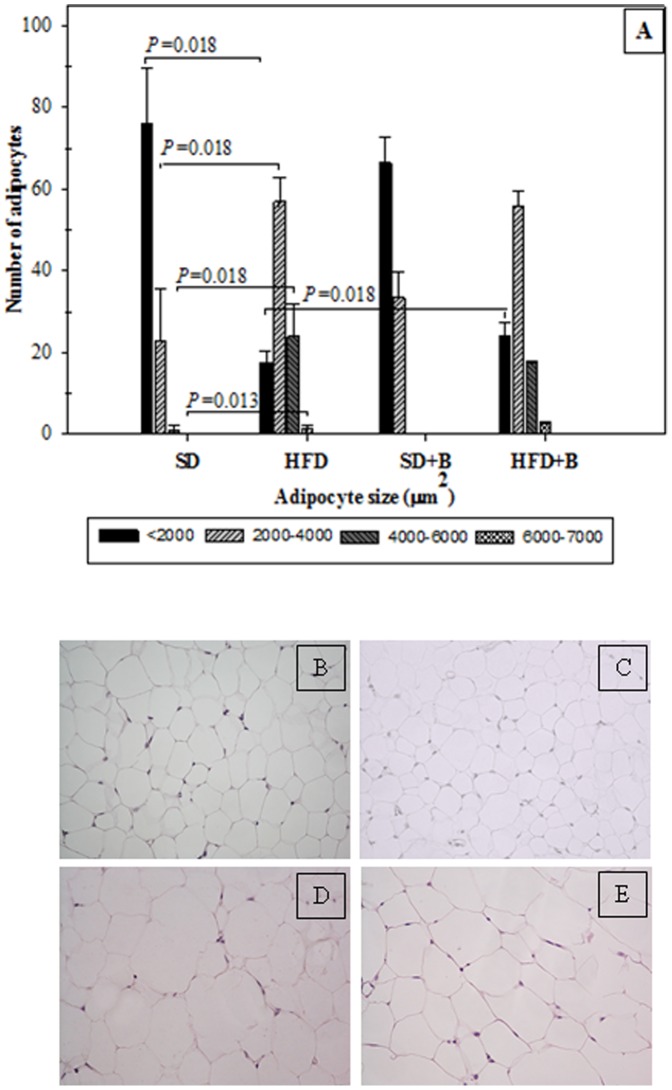
Distribution of adipocyte size in epididymal adipose tissue in control mice and mice with high fat diet-induced obesity, administered or not *B. uniformis* CECT 7771. SD: standard diet group (control) (n = 6); SD+B: standard diet group receiving a daily dose of 5.0×10^8^ CFU *B. uniformis* CECT 7771 by gavage for 7 weeks (n = 6); HFD: high fat diet group (n = 6); HFD+B: high fat diet group receiving a daily dose of 5.0×10^8^ CFU *B. uniformis* CECT 7771 by gavage for 7 weeks (n = 6). Adipocyte cell sizes were expressed as area ranges and were the following: <2000, 2000–4000, 4000–6000 and 6000–7000 µm^2^. Data are expressed as means ± SD and statistically significant differences are established at *P*<0.05. Photomicrographs 20X of representative HE-stained slides are shown. (B) SD group, (C) SD+B group, (D) HFD group and (E) HFD+B group.

The effects of the intervention on adipocyte size on epididimal adipose tissue are shown in Figure 4. HFD induced a significant increase in adipocyte size in the following ranges 2000–4000, 4000–6000 and 6000–7000 µm^2^, and reduced those of size <2000 µm^2^. The administration of *B. uniformis* CECT 7771 in SD-fed mice did not induce significant changes in adipocyte size, while a significant increase in the number of small adipocytes (<2000) was observed in HFD-fed mice.

**Figure 5:**
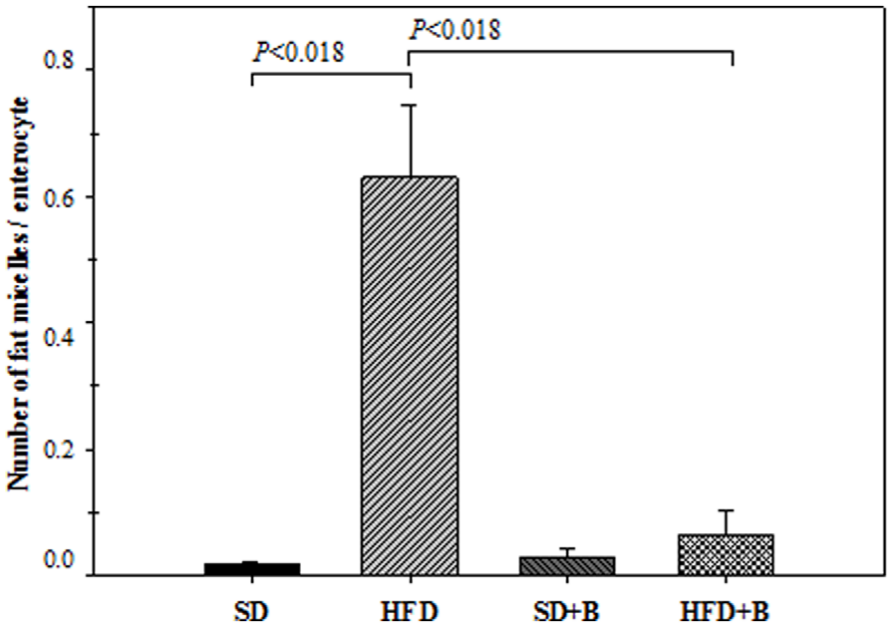
Number of fat micelles per enterocyte in control mice and mice with high fat diet-induced obesity, administered or not *B. uniformis* CECT 7771. SD: standard diet group (control) (n = 6); SD+B: standard diet group receiving a daily dose of 5.0×10^8^ CFU *B. uniformis* CECT 7771 by gavage for 7 weeks (n = 6); HFD: high fat diet group (n = 6); HFD+B: high fat diet group receiving a daily dose of 5.0×10^8^ CFU *B. uniformis* CECT 7771 by gavage for 7 weeks (n = 6). The relation fat micelles/enterocyte was determined in 100 cells from two sections of the small intestine of each mouse by counting ten 100X light microscope fields. Data are expressed as means ± SD and statistically significant differences are established at *P*<0.05.

The effects of the HFD and the administration of the bacterial strain on the number of fat micelles per enterocyte, which indicate dietary fat absorption, are shown in Figure 5. The HFD induced a significant increase in fat micelles in enterocytes, whereas the administration of *B. uniformis* CECT 7771 reduced these numbers. In the SD group, no significant changes were observed due to intervention with the aforementioned bacterium.

### Macrophage Functionality

**Figure 6:**
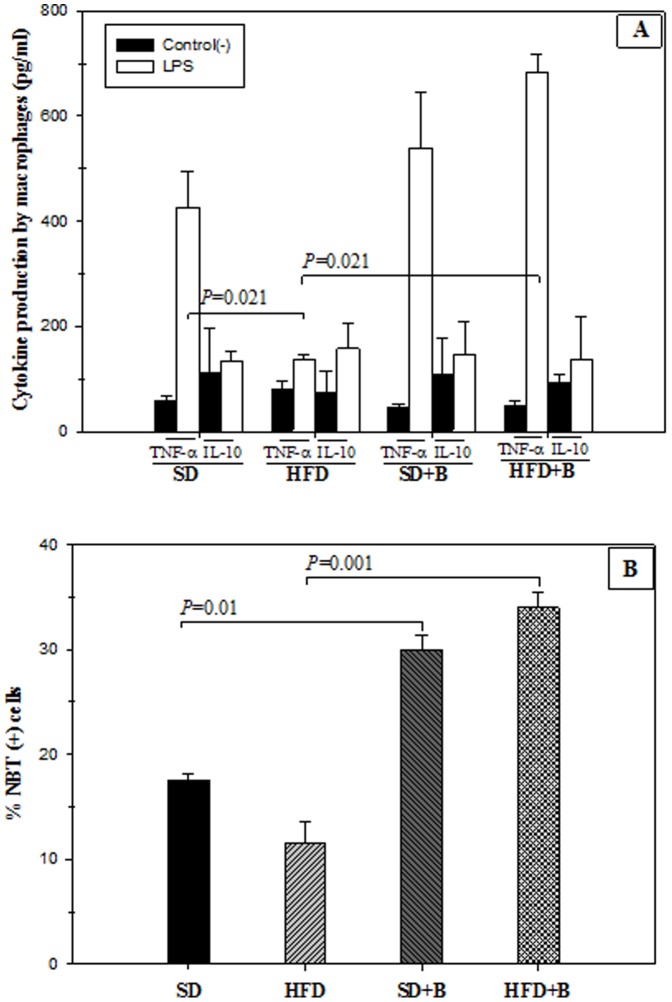
Cytokine production in LPS-stimulated peritoneal macrophages and phagocytosis function of control mice and mice with high-fat diet induced obesity, administered or not *B. uniformis* CECT 7771. SD: standard diet group (control) (n = 6); SD+B: standard diet group receiving a daily dose of 5.0×10^8^ CFU *B. uniformis* CECT 7771 by gavage for 7 weeks (n = 6); HFD: high fat diet group (n = 6); HFD+B: high fat diet group receiving a daily dose of 5.0×10^8^ CFU *B. uniformis* CECT 7771 by gavage for 7 weeks (n = 6). In the cytokine production study, peritoneal macrophages were stimulated with purified lipopolysaccharide (LPS) from *S. enterica* serotype Typhimurium (Figure 6A). Non-stimulated peritoneal macrophages were evaluated as controls of basal cytokine levels. In the phagocytosis study (Figure 6B), evidence of oxygen-radical production by macrophages was determined by the NBT test after *in vitro* interaction with a bacterial extract. Figure 6A: TNF- α and IL-10 cytokines produced by LPS-stimulated macrophages; Figure 6B: % NBT (+) cells. Data are expressed as mean and standard deviation of duplicate measurements determined in two independent experiments. Statistically significant differences of data are established at *P*<0.05.

The results of cytokine production by LPS-stimulated peritoneal macrophages from SD- and HFD-fed mice with and without B. *uniformis* CECT 7771 supplementation are shown in Figure 6. Obesity induced by a HFD led to a decrease in cytokine TNF-α production by peritoneal macrophages stimulated with LPS compared to macrophages of SD-fed mice (Figure 6A). The administration of *B. uniformis* CECT 7771 significantly increased the ability of LPS-stimulated macrophages to produce TNF-α in HFD-fed mice but not in SD-fed mice (Figure 6A). The HFD did not affect the ability of LPS-stimulated macrophages to produce the anti-inflammatory cytokine IL-10 and this feature was not modified by the administration of *B. uniformis* CECT 7771 (Figure 6A). The oxidative burst in peritoneal macrophage after uptake of a microbial extract was also studied to analyse effects on phagocytosis function (Figure 6B). The results indicated that the oral administration of *B. uniformis* CECT 7771 stimulated this function in macrophages in both SD- and HFD-fed mice (Figure 6B).

### Dendritic Cell (DC) Functionality

**Figure 7:**
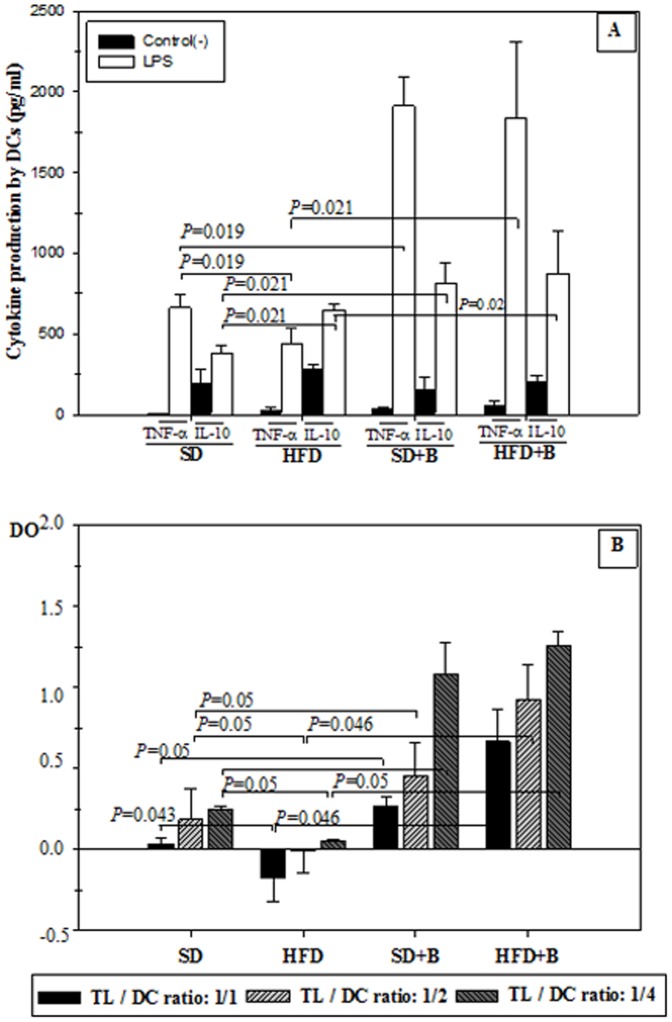
Influence of LPS stimuli on cytokine production and activation of T-lymphocyte proliferation by dendritic cells (DCs) generated from control mice and mice with high-fat diet induced obesity, administered or not *B. uniformis* CECT 777. SD: standard diet group (control) (n = 6); SD+B: standard diet group receiving a daily dose of 5.0×10^8^ CFU *B. uniformis* CECT 7771 by gavage for 7 weeks (n = 6); HFD: high fat diet group (n = 6); HFD+B: high fat diet group receiving a daily dose of 5.0×10^8^ CFU *B. uniformis* CECT 7771 by gavage for 7 weeks (n = 6). In the cytokine production study, DCs were stimulated with purified lipopolysaccharide (LPS) from *S. enterica* serotype Typhimurium (Figure 7A). Non-stimulated DCs were evaluated as controls of basal cytokine levels. In the lymphocyte proliferation study (Figure 7B), matured DCs were used for priming a T-cell proliferative response at the following LT/CD ratios: 1∶1, 1∶2, 1∶4. Lymphocyte proliferation was measured with the cell proliferation ELISA BrdU-colorimetric assay. Figure 7A: TNF- α and IL-10 cytokines produced by LPS-stimulated CDs; Figure 7B: Lymphocyte proliferation. Data are expressed as means ± SD of duplicate measures determined in two independent experiments. Statistically significant differences of data are established at *P*<0.05.

The results of cytokine production by LPS-stimulated DC from SD- and HFD-fed mice, with and without *B. uniformis* CECT 7771 supplementation are shown in Figure 7A. The administration of *B. uniformis* CECT 7771 to obese and lean mice increased the ability of DC to produce TNF-α in response to LPS stimulation, which was significantly reduced by the HFD (Figure 7A). In HFD-fed mice, IL-10 production by LPS-stimulated DCs was significantly increased. The administration of *B. uniformis* CECT 7771 increased IL-10 values even more in HFD-fed mice and also stimulated this cytokine production in SD-fed mice (Figure 7A).

The results of the influence of HFD-induced obesity and oral administration of *B. uniformis* CECT 7771 on the ability of matured DCs to priming a T cell proliferative response are shown in Figure 7B. DCs from SD-fed mice were able to induce a significant increase in T cell proliferation in comparison to T cells alone (data not shown) in all the examined DC:T cell ratios. The HFD impaired the capacity of DCs to induce a T cell proliferation response, but this function was restored by *B. uniformis* CECT 7771 administration, and the strongest effects were obtained at 1∶4 DC:T ratio. This effect was also significant for SD-fed mice.

### Microbiota Composition and Inflammatory Properties

The composition of the faecal microbiota in SD- and HFD-fed mice is shown in Table 4. The HFD led to reductions in the gene copy numbers of most of the bacterial groups analysed, including *Lactobacillus*, *C. coccoides* and *C. leptum* groups and the genus *Bifidobacterium*. This diet also caused increases in gene copy numbers of members of the *Enterobacteriaceae* family. In HFD-fed mice, *B. uniformis* CECT 7771 administration increased the gene copy numbers of the genera *Bacteroides* and *Bifidobacterium* and the group *C. coccoides* and reduced those of members of the *Enterobacteriaceae* family, partially restoring the alteration of the microbiota associated with the HFD. In SD fed mice, the administration of the strain led to an increase in the gene copy numbers of total bacteria the genera *Bacteroides* and *Bifidobacterium* and the group *C. leptum.* The numbers of the *C. coccoides* group followed the same trend, but the differences were not significant.

**Table 4:** Microbiota composition of stool samples from different mouse groups analysed by quantitative PCR^a^.

Bacterial group	Experimental groups						
	SD	HFD		SD+B		HFD+B	
	aMedian (IQR)	aMedian (IQR)	p- value^b^	aMedian (IQR)	p- value^c^	aMedian (IQR)	p- value^d^
Total bacteria	10.8 (10.6–11.1)	10.5 (10.3–10.8)	0.092	11.4 (11.3–11.6)	0.010*	11.0 (10.7–11.2.)	0.629
*Lactobacillus* group	9.9 (9.4–10.5)	9.4 (9.2–9.5)	0.040*	9.6 (9.3–9.8)	0.470	9.7 (9.5–10.1)	0.936
*Bacteroides* spp.	8.4 (8.3–8.6)	8.7 (8.3–9.0)	0.674	9.3 (9.1–9.5)	0.004*	9.0 (8.8–9.3)	0.016*
*Bifidobacterium* spp.	7.1 (6.8–7.2)	6.0 (5.9–6.3)	0.004*	8.1 (7.9–8.3)	0.013*	7.5 (7.0–7.7)	0.004*
*C. leptum* group	8.4 (8.3–8.6)	7.6 (7.5–7.7)	0.004*	9.6 (9.4–9.8)	0.004*	8.5 (8.1–8.7)	0.936
*C. coccoides* group	9.1 (8.6–9.3)	8.4 (8.2–8.5)	0.016*	9.9 (9.4–10.0)	0.054	9.6 (9.4–9.7)	0.036*
*Enterobacteriaceae*	7.3 (7.2–7.7)	8.1 (7.8–8.2)	0.019*	8.1 (7.6–8.2)	0.052	7.9 (7.5–8.0)	0.029*

SD: standard diet group (control) (n = 6); SD+B: standard diet group receiving a daily dose of 5.0×10^8^ CFU *B. uniformis* CECT 7771 by gavage for 7 weeks (n = 6); HFD: high fat diet group (n = 6); HFD+B: high fat diet group receiving a daily dose of 5.0×10^8^ CFU *B. uniformis* CECT 7771 by gavage during 7 weeks (n = 6).

a Data are expressed as median of log gene copy numbers of each bacterial group per gram of stools.

b Significant differences in log gene copy numbers of specific bacterial groups between SD and HFD mouse groups.

c Significant differences in log gene copy numbers of specific bacterial groups between SD and SD+B mouse groups.

d Significant differences in log gene copy numbers of specific bacterial groups between HFD and HFD+B groups.

* Significant differences were established at *P*<0.05 by using Mann–Whitney U-test.

**Figure 8:**
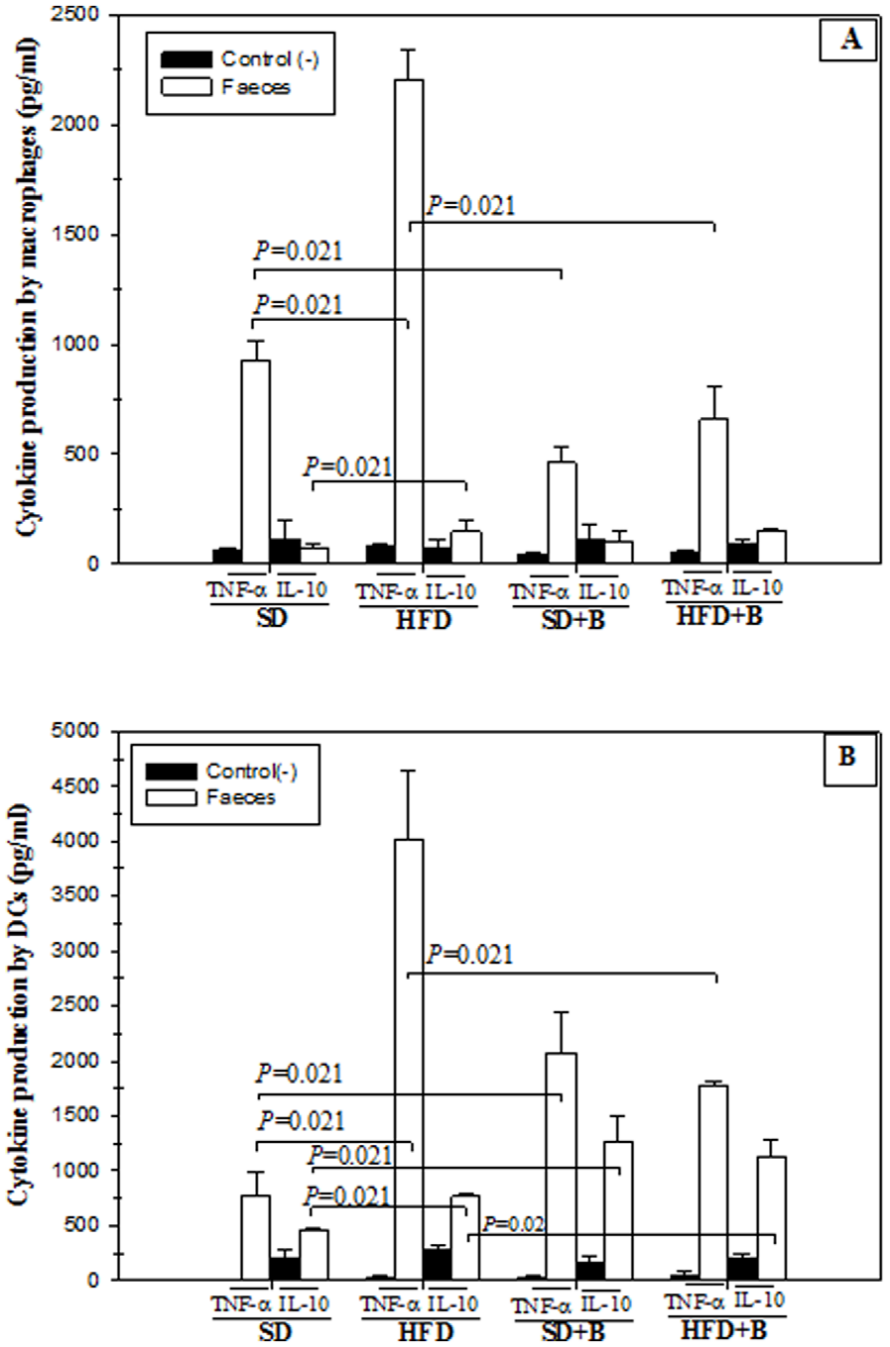
Influence of stool samples from mice fed standard diet or high-fat diet, supplemented or not with *B. uniformis* CECT 7771, on cytokine production by peritoneal macrophages and dendritic cells (DCs) from control mice. SD: standard diet group (control) (n = 6); SD+B: standard diet group receiving a daily dose of 5.0×10^8^ CFU *B. uniformis* CECT 7771 by gavage for 7 weeks (n = 6); HFD: high fat diet group (n = 6); HFD+B: high fat diet group receiving a daily dose of 5.0×10^8^ CFU *B. uniformis* CECT 7771 by gavage for 7 weeks (n = 6). In the cytokine production study, peritoneal macrophages (Figure 8A) and DCs (Figure 8B) were stimulated with stool stimuli. Non-stimulated peritoneal macrophages and DCs were evaluated as controls of basal cytokine levels. Figure 8A: TNF- α and IL-10 cytokines produced by stool-stimulated peritoneal macrophages; Figure 8B: TNF- α and IL-10 cytokines produced by stool-stimulated CDs. Data are expressed as means ± SD of duplicate measures determined in two independent experiments. Statistically significant differences of data are established at *P*<0.05.

To evaluate whether these changes in the microbiota could modify the inflammatory signals coming from the gut in the different mouse groups, the ability of faecal samples to induce the production of cytokines by immunocompetent cells *in vitro* was evaluated (Figure 8A and 8B). Stool samples from HFD-fed mice induced a higher production of TNF-α than those from SD-fed mice by macrophages (Figure 8A) and DCs (Figure 8B), indicating that the HFD induced an increase in the pro-inflammatory signals coming from the gut. The administration of *B. uniformis* CECT 7771 significantly reduced the production of this pro-inflammatory cytokine in macrophages stimulated with stools from both mouse groups (Figure 8A), and in DCs stimulated with stools from the HFD group. Therefore, this bacterial strain seems to have ability to reduce the inflammatory properties of the gut content, although confirmatory studies measuring additional inflammatory markers in the gut would be required to draw definitive conclusions.

In macrophages (Figure 8A) and DCs (Figure 8B), stool samples of HFD-fed mice induced significantly higher production of the anti-inflammatory cytokine IL-10 than those from SD-fed mice, which could be due to the activation of regulatory mechanisms to counteract other inflammatory signals inducing also TNF-α. Stool samples from both SD and HFD mouse groups administered *B. uniformis* CECT 7771 induced a significant increase in IL-10 production by DCs, suggesting an increase in the anti-inflammatory signals emanating from the gut (Figure 8B). These *B. uniformis* CECT 7771-related effects were not detected in macrophages (Figure 8A).

## Discussion

This study has provided direct evidence of the metabolic and immune effects of *B. uniformis* CECT 7771 in a murine model of diet-induced obesity. Previous scientific research was controversial and had only established associations between either a lean phenotype or weight loss with increased intestinal numbers of the phylum Bacteroidetes phylum, or the groups *Bacteroides-Prevotella* or *Bacteroides fragilis* [9], [11]–[20]. The selection of the specific strain tested was based on its lower inflammatory potential *in vitro* on macrophages compared to strains belonging to other intestinal *Bacteroides* spp. This trait could be relevant in the context of obesity since it is considered a chronic inflammatory disorder, largely mediated by macrophage infiltration in the adipose tissue [31]–[33]. Our study demonstrated that *B. uniformis* CECT 7771 induced low TNF-α production and the highest IL-10 production in comparison with other strains of *Bacteroides* spp. Our recent studies also demonstrated that the prevalence of *B. uniformis* CECT 7771 in the gut of infants is favoured by breast-feeding [22] and breast-feeding seems to protect against later development of obesity as compared to formula feeding [34]–[35]. Considering these data altogether led us to hypothesise that the oral administration of *B. uniformis* CECT 7771 could exert beneficial effects in an obesity model as reported in this study.

In our mouse obesity model, *B. uniformis* CECT 7771 administration induced significant modifications in total body weight gain by the end of intervention, although differences in adipose tissue weight were not significant, probably due to the limited duration of the trial. However, *B. uniformis* CECT 7771 increased the number of small adipocytes in obese mice, which could precede fat weight reduction. In contrast, colonisation of germ-free mice by *B. thetaiotaomicron* and *Methanobrevibacter smithii* augmented *de novo* lipogenesis and adiposity [36]. In this context, it was proposed that gut colonisation by the conventional microbiota or by specific commensal bacteria (e.g. *B. thetaiotaomicron*) provides the hydrolases necessary to utilise complex polysaccharides and the resulting products are absorbed or metabolised to short-chain fatty acids. Subsequently, the latter are delivered to the liver and converted to triacylglycerols and, then, part of these *de novo* synthesised lipids are deposited in adipocytes [37]–[38]. In addition, it was considered that the gut microbiota could reduce the fasting-induced adipose factor (Fiaf, also known as angiopoietin-like protein-4), a secreted lipoprotein lipase (LPL) inhibitor [8], promoting storage of fatty acids released by the LPL in the host adipose tissue, but this mechanism was not confirmed in a latter study [39]. Although the animal models used in our and previous studies are not comparable, the results suggest that different *Bacteroides* spp. might exert different effects on energy balance and lipid storage.

In obese subjects there is also an increased flux of free fatty acids to the liver due to their excessive accumulation in the adipose tissue and to the inability of insulin to suppress lipolysis in adipocytes due to insulin resistance, which leads to steatosis or fatty liver disease [40]–[41]. In our study, the administration of *B. uniformis* CECT 7771 significantly reduced steatosis in obese animals and, interestingly, also reduced liver cholesterol and triglyceride accumulation. In obese mice, liver steatosis and adipocyte hypertrophy is reported to be related to an increased energy input via intestinal lipid absorption, which is transported in the form of chylomicrons to peripheral tissues [42]. Therefore, we also analysed the number of fat micelles per enterocyte, which partly represents fat absorbed from the diet. *B. uniformis* CECT 7771 administration significantly reduced the fat micelles in enterocytes, particularly in obese mice, indicating that this is a mechanism by which this bacterial strain exerts a positive effect on liver steatosis and serum lipids. Other studies also demonstrated that specific lactobacilli or bifidobacterial strains might reduce the absorption of dietary fat, but this is the first evidence that a *Bacteroides* strain exerts this effect depending on the diet [40], [42], [43]. However, the involvement of other mechanisms, such as reduction of endogenous biosynthesis or reduction of lipid uptake by hepatocytes, cannot be disregarded [44].

Dyslipidaemia is also a frequent feature of obese subjects, with the most common being hypertriclyceridaemias and hypercholesterolaemias [45], [46], which are also induced by HFD in mice. In this study, HFD-fed mice showed higher values of serum cholesterol and triglyceride levels than SD-fed mice. *B. uniformis* CECT 7771 administration reduced serum cholesterol and triglyceride levels in HFD-fed mice. These effects could also be partially related to a reduction of dietary fat absorption and possibly to modulation of the expression of genes and proteins involved in lipid homeostasis in the gut and liver, as indicated previously.

Hyperglycaemia and insulin resistance are also frequently associated with obesity in humans [1]–[2]. This feature was also reproduced in our mice obesity model, which showed increased fasting glucose levels and reduced glucose tolerance. However, the administration of *B. uniformis* CECT 7771 significantly improved the response to an oral glucose challenge and reduced the fasting glycaemia in parallel to insulin, suggesting an improvement in glucose metabolism and insulin sensitivity. Comparisons between germ-free and conventional mice indicated that the commensal microbiota, as a whole, induced hyperglycaemia and insulin resistance [44] while administration of antibiotics causing a reduction in the intestinal bacterial load improved oral glucose tolerance in *ob*/*ob* and HFD-fed mice [47], [48]. The adverse effects attributed to the microbiota were related to a reduction of AMPK activity and, therefore, reduced insulin-stimulated glucose transport in muscles [38] and to a reduction of inflammatory signals coming from the gut, such as LPS from Gram-negative bacteria and its correlation with intestinal and plasma TNF-α levels [49]. Nevertheless, our present and previous studies indicated that intervention in the gut ecosystem with specific strains may improve diet-induced insulin sensitivity and glucose tolerance above the commensal microbiota effects [40].

Obesity often manifests with hyperleptinemia, associated with leptin resistance, leading to different central and peripheral adverse effects, including increased hunger and reduced energy expenditure as well as increased lipid accumulation [50]. *B. uniformis* CECT 7771 administration also led to reduced leptin levels in obese mice, which could be indicative of an improvement in leptin function or sensitivity, leading to lower leptin production [51]. These improved leptin levels could be related to improvements in glucose tolerance and reduced serum concentration since leptin favours insulin function [52]. In addition, the reduction of serum leptin levels could also be related to increases in smaller adipocytes and reduced liver steatosis in HFD-fed mice supplemented with the bacterial strain due to the role of leptin in fat accumulation in peripheral tissues [53].

Our study also showed that *B. uniformis* CECT 7771 administration improves immune function of macrophages and DCs, which is particularly important in obese mice. It is known that macrophage function is impaired in obesity, showing reduced phagocytic capacity and oxidative burst, which has been linked to increased susceptibility to infections of obese subjects [54], [55]. In our study, phagocytic function of macrophages was slightly reduced by the HFD, in accordance with others authors [56]. However, *B. uniformis* CECT 7771 administration stimulated the oxidative burst of macrophages in both HFD and SD-fed mice. Our study also demonstrates that *B. uniformis* CECT 7771 administration improved the ability of macrophages and DCs to produce cytokines in response to a pathogenic bacterial stimulus (LPS). *B. uniformis* CECT 7771 also restored the capacity of DCs to present antigens and stimulate T lymphocyte proliferation. A functional deficiency of DCs in *ob/ob* mice has also been described previously, suggesting that this damage could be generalised to the more frequent forms of obesity [41]. Altogether, our data indicates that *B. uniformis* CECT 7771 can improve innate and adaptive defence mechanisms against infections in diet-induced obesity.

This study also confirms that diet has a tremendous impact on the intestinal microbiota composition and supports the hypothesis that its modulation through the use of dietary strategies could ameliorate the metabolic and immune dysfunctions associated with imbalanced diets. The HFD reduced gene copy numbers of Gram-positive bacteria, including *Bifidobacterium* spp. and *C. coccoides* group, in accordance with previous studies [49]. Our study also demonstrates that the HFD increased the gene copy numbers of enterobacteria, while *B. uniformis* CECT 7771 slightly reduced these numbers and increased those of *C. coccoides* group, *Bifidobacterium* spp. and *Bacteroides* spp. Furthermore, the *B. uniformis* CECT 7771-related changes in microbiota contributed to reducing the gut inflammatory signals, which could affect other peripheral tissues involved in obesity. In this context, endotoxins (LPS) from enterobacteria, present in the gut, have been shown to play an important role in the development of insulin resistance and non-alcoholic fatty liver disease [57]. In *ob/ob* mice, reductions of intestinal *E. coli* numbers by antibiotic treatment were associated with the reduction of metabolic endotoxaemia and inflammatory status as well as with improvements in insulin resistance [58]. Another recent study also reported that positive effects of prebiotics (oligofructose and inulin mixture) on body weight and fat and related metabolic parameters could be due to their ability to increase numbers of both bacteroides and bifidobacteria in the gut microbiota of obese JCR:LA-cp rats [13]. These changes were related to modifying the expression of anorexigenic and orexigenic peptides, but the role of each bacterial group on such effects remains unclear.

In conclusion, the results support the hypothesis that the *B. uniformis* CECT 7771 modulates the metabolic and immune dysfunction induced by HFD at least in mice. The study provides direct evidence of the potential beneficial roles played by *B. uniformis* CECT 7771 in obesity, bringing us one step ahead of the mere associations inferred from previous observational studies. Therefore, the use of dietary strategies targeting the gut ecosystem may be an additional tool to control metabolic disorders. However, further studies are required to support this hypothesis and to reveal plausible mechanisms of action of the specific bacterial strain used in this preclinical study. Furthermore, safety issues should also be addressed before proposing the possible use of the strain tested in humans.
